# High dose intravenous iron, mineral homeostasis and intact FGF23 in normal and uremic rats

**DOI:** 10.1186/1471-2369-14-281

**Published:** 2013-12-27

**Authors:** Eva Gravesen, Jacob Hofman-Bang, Maria L Mace, Ewa Lewin, Klaus Olgaard

**Affiliations:** 1 Nephrological Department P, Rigshospitalet, University of Copenhagen, P 2132, 9 Blegdamsvej, Copenhagen DK 2100, Denmark; 2 Nephrological Department B, Herlev Hospital, Copenhagen, Denmark

**Keywords:** Iron, FGF23, Phosphate, Chronic kidney disease

## Abstract

**Background:**

High iron load might have a number of toxic effects in the organism. Recently intravenous (iv) iron has been proposed to induce elevation of fibroblast growth factor 23 (FGF23), hypophosphatemia and osteomalacia in iron deficient subjects. High levels of FGF23 are associated with increased mortality in the chronic kidney disease (CKD) population. CKD patients are often treated with iv iron therapy in order to maintain iron stores and erythropoietin responsiveness, also in the case of not being iron depleted. Therefore, the effect of a single high iv dose of two different iron preparations, iron isomaltoside 1000 (IIM) and ferric carboxymaltose (FCM), on plasma levels of FGF23 and phosphate was examined in normal and uremic iron repleted rats.

**Methods:**

Iron was administered iv as a single high dose of 80 mg/kg bodyweight and the effects on plasma levels of iFGF23, phosphate, Ca2+, PTH, transferrin, ferritin and iron were examined in short and long term experiments (n = 99). Blood samples were obtained at time 0, 30, 60, 180 minutes, 24 and 48 hours and in a separate study after 1 week. Uremia was induced by 5/6-nephrectomy.

**Results:**

Nephrectomized rats had significant uremia, hyperparathyroidism and elevated FGF23. Iron administration resulted in significant increases in plasma ferritin levels. No significant differences were seen in plasma levels of iFGF23, phosphate and PTH between the experimental groups at any time point within 48 hours or at 1 week after infusion of the iron compounds compared to vehicle.

**Conclusions:**

In non-iron depleted normal and uremic rats a single high dose of either of two intravenous iron preparations, iron isomaltoside 1000, and ferric carboxymaltose, had no effect on plasma levels of iFGF23 and phosphate for up to seven days.

## Background

Intravenous (iv) iron supplementation is an integrated part of the management of anemia in patients with chronic kidney disease (CKD). Treatment of anemia in CKD requires in many instances erythropoietin (EPO). To ensure that full benefit from EPO is obtained most patients require iron supplement during the treatment, even when they are not iron depleted [1]. Intravenous iron administration has been shown to reduce the need for EPO and to make anemia treatment more cost effective [2-4]. However, excessive iron load is potentially harmful. Evidence from experimental studies have shown that iron excess can exert cytotoxicity, exacerbate infection, inhibit neutrophil activity, enhance oxygen radical generation, promote arteriosclerosis and increase mortality [5-8]. Recently a link between fibroblast growth factor 23 (FGF23) and iron homeostasis has been proposed [9-14], potentially adding a further negative effect to those reported from iv administration of iron [5-8,15].

During recent years the bone derived phosphaturic hormone, FGF23 and its co-receptor, Klotho, have been extensively studied and their role in phosphate homeostasis is now well established, although many questions regarding the regulation of FGF23 and Klotho still remain unanswered [16]. It is well documented that disturbed phosphate metabolism plays a critical role in the pathophysiology of uremia and that hyperphosphatemia is an important factor in the development of vascular calcification in CKD [17]. Plasma FGF23 increases very early in the development of CKD and is associated with cardiovascular mortality. In dialysis patients FGF23 levels are extremely elevated. FGF23 itself has been shown to mediate off-target direct end-organ toxicity in the heart promoting cardiomyocyte hypertrophy. Elevated FGF23 may represent a specific factor in the adverse cardiovascular outcome and is not only a parameter of disturbed phosphate homeostasis in CKD [18-21]. A further iron-triggered augmentation of the already increased FGF23 levels in CKD patients might as such theoretically contribute to the potential negative effects of FGF23 on mortality [20].

In non-CKD patients iv iron was shown to induce increased FGF23 secretion [13,14]. Recent data suggest that iron deficiency may stimulate FGF23 transcription [19,22,23]. A more recent study by Wolf et al. [11] compared the effect of a single dose of ferric carboxymaltose (FCM) or iron dextran on intact FGF23 (iFGF23) and C-terminal FGF23 (cFGF23) levels in iron-deficient, anemic women with normal kidney function. In accordance with prior reported results they found that iron deficiency was associated with markedly increased cFGF23 levels. After administration of both FCM and iron dextran cFGF23 fell significantly, while a transient increase in iFGF23 levels was observed within the first 24 hours in the FCM group [11]. This underlines the importance of designing studies on iron effects/toxicity with the inclusion of several measurements, as the plasma concentrations of the biologically active compound, iFGF23, and its inactive fragment, cFGF23 might fluctuate and respond differently. In the same study the authors proposed the possibility that induction of non-skeletal sources of FGF23 by iron treatment in iron deficient subjects may theoretically take place. The complex relationship between iron status and FGF23 regulation might potentially reflect both disturbed bone remodeling in iron deficiency [24], disturbed iron sensing and signaling and adaptive responses to iron deficiency [25]. It is therefore of importance to investigate the effect of iv iron not only in iron depleted subjects, but also in normal condition.

In CKD, Takeda et al. examined the effects of iv iron on iFGF23 in hemodialysis patients [12] and found a significant increase in iFGF23 after 40 mg of saccharated iron three times weekly over three months. This was not accompanied by hypophosphatemia due to absence of kidney function, while a significant, but transient decrease in plasma PTH was demonstrated, further stressing the importance of also examining the effect of iv iron on other parameters of mineral homeostasis, even though focus of the study mainly is on the relationship between iron and FGF23. The susceptibility to iron mediated toxicity can differ between normal and CKD conditions, as previously demonstrated [5] and demands for examinations not only at normal GFR, but also in models of CKD.

The recent evidence for an effect of iv iron on the rise of FGF23 levels is generated from studies on iron depleted subjects [11,13,14,22,23,26]. However, iv iron treatment in the CKD population is also often routinely provided to iron repleted individuals in order to maintain iron stores and EPO responsiveness, and as such it is of importance to study whether iv iron administration influences FGF23 and mineral homeostasis in the iron repleted condition.

The aim of the present study therefore was in non-iron deficient normal and uremic rats to examine the short and long-term effects of a single high intravenous dose of two different iron preparations, iron isomaltoside 1000 (IIM) and ferric carboxymaltose (FCM) on plasma FGF23 levels and other parameters of the mineral homeostasis.

## Methods

### Animals

Adult male Wistar rats, 250 g (Taconic A/S, Ejby, Denmark) were housed in a temperature-controlled environment with a 12-hour light/dark circle with free access to food and water. The experimental studies were performed in accordance with Danish law and approved by the Animal Experiments Inspectorate, the Ministry of Food, Agriculture and Fisheries, Denmark.

### Uremia

Chronic renal failure (CRF) was induced by one-step 5/6 nephrectomy, as previously described [27]. Rats were anaesthetized with hypnorm/midazolam (Panum Institute, Copenhagen, Denmark), and were given carprofen (Rimadyl, Pfizer, Copenhagen, Denmark) subcutaneously as pain relief for the following three days. CRF rats (n = 54) were fed a high phosphorus diet containing 1.4% phosphorus, 0.9% calcium, per kg (Altromin, Spezialfutter GmbH & Co., KG, Germany) in order to induce severe uremia and hyperparathyroidism. Duration of uremia was 8 weeks. Normal rats (n = 45) were fed a standard diet containing 0.9% calcium, 0.7% phosphorus, 165 mg iron and 600 IU vitamin D per kg diet (Altromin, Germany).

### Iron preparations and administration

Iron isomaltoside 1000 (IIM) (Monofer, Pharmacosmos, Holbaek, Denmark) is a chemical modification of isomalto-oligosaccharides that consists predominantly of 3–5 non-branched glucose units which bind iron within the matrix. Ferric carboxymaltose (FCM) (Ferinject, Vifor Pharma, Glattbrug, Switzerland) contains iron in a stable ferric state as a complex with a carbohydrate polymer. The dose of the two iron preparations given in this trial was 80 mg/kg body weight. The maximal recommended single dose in humans of IIM is 20 mg/kg body weight and a total of 1000 mg for FCM. The iron was given intravenously as a single dose of either IIM or FCM. The iron preparations were diluted in sterile isotonic sodium chloride in a total volume of 1 ml. The vehicle group received 1 mL of sodium chloride. IIM, FCM or vehicle was injected into the femoral vein after free dissection under stereomicroscope.

### Design

The studies were performed both as a short-term (48 hour) intensive study and a long- term (1 week) study to assess the effects of a single intravenous injection of IIM or FCM on plasma FGF23 and other biochemical parameters in normal and uremic rats.

In the 48-hour studies blood samples were obtained at baseline and 30, 60, 120 and 180 minutes and again after 24 and 48 hour. In the 1-week studies blood samples were obtained at baseline and after 1 week. The following experimental groups were examined, using a total of 99 rats:

Group 1: Normal rats, (48 hours) given vehicle (n = 9)

Group 2: Normal rats, (48 hours) given IIM (n = 8)

Group 3: Normal rats, (48 hours) given FCM (n = 9)

Group 4: Uremic rats, (48 hours) given vehicle (n = 11)

Group 5: Uremic rats, (48 hours) given IIM (n = 10)

Group 6: Uremic rats, (48 hours) given FCM (n = 14)

Group 7: Normal rats, (1 week) given vehicle (n = 6)

Group 8: Normal rats, (1 week) given IIM (n = 7)

Group 9: Normal rats, (1 week) given FCM (n = 6)

Group 10: Uremic rats, (1 week) given vehicle (n = 6)

Group 11: Uremic rats, (1 week) given IIM (n = 7)

Group 12: Uremic rats, (1 week) given FCM (n = 6)

As we observed that plasma levels of cFGF23 increased during the short-term experimental conditions, we decided to examine the performance of C-terminal and intact FGF23 without any intervention and further to examine the potential effect of two different anesthetics on cFGF23 levels. Blood samples were obtained at 0, 60 and 120 minutes in:

Group 13: Normal rats, no sedation (n = 4)

Group 14: Normal rats, given pentobarbital anesthesia (n = 4)

Group 15: Normal rats, given hypnorm/midazolam anesthesia (n = 4)

### Biochemistry

Plasma iFGF23 was measured by an intact FGF23 ELISA assay (Kainos Laboratories, Tokyo, Japan), intra-assay coefficient of variation of 2.5% and inter-assay coefficient of variation of 5% in our lab. Plasma cFGF23 was measured by a C-terminal FGF23 ELISA assay (Immutopics, San Clemente, CA, USA) intra-assay coefficient of variation of 5.4% and inter-assay coefficient of variation of 5.2%. Plasma PTH was measured by a rat bioactive intact PTH ELISA assay (Immunotopics, San Clemente, CA, USA) with an intra- assay variation of 4% and inter-assay variation of 9% [28]. Plasma ferritin and plasma transferrin were both measured by ELISA assays (Alpco, Salem, New Hampshire, USA). Plasma phosphate, urea and creatinine were analyzed by Vitros 250 (Ortho-Clinical Diagnostics, Raritan, NJ, USA). Plasma Ca^2+^, K^+^, and Na ^+^ by ABL 505 (Radiometer, Copenhagen, Denmark). Plasma iron was measured at Department of Biochemistry, Rigshospitalet, Copenhagen, DK.

### Statistics

All data are presented as mean ± SEM. Differences between multiple groups were analyzed using one-way ANOVA followed by Bonferroni’s *post hoc* test. When comparing multiple groups at different time points two-way ANOVA was used. Two-sided t-test was used when comparing only two groups with normal distribution. P < 0.05 was considered statistically significant. The analyses were performed using Graph Pad Prism version 4.

## Results

### Induction of uremia and iron administration

Uremia and severe secondary hyperparathyroidism were successfully induced by 5/6- nephrectomy and high-phosphate diet, Table 1. Uremic animals had significantly elevated levels of plasma creatinine, urea and iFGF23 as well as significantly elevated plasma phosphate and PTH as compared to normal rats (p < 0.0001). Basal plasma ferritin and iron levels were higher in the uremic group (p < 0.05).

**Table 1:** Baseline plasma biochemistry

**Plasma parameters**	**Normal rats**	**Uremic rats**
**n**	**45**	**54**
FGF23, pg/mL	118 ± 10	1624 ± 136***
PTH, pg/mL	59 ± 11	1592 ± 194***
Ferritin, ng/mL	423 ± 78	698 ± 87*
Iron, μmol/L	16.3 ± 2.5	26.6 ± 4.0*
Transferrin, g/L	1.6 ± 0.1	1.4 ± 0.1
Creatinine, μmol/L	25.8 ± 0.5	76.9 ± 3.9***
Urea, mmol/L	7.6 ± 0.2	22.4 ± 0.9***
Ca^2+^, mmol/L	1.37 ± 0.01	1.09 ± 0.02***
P, mmol/L	1.80 ± 0.06	2.88 ± 0.11***
K, mmol/L	5.0 ± 0.1	5.4 ± 0.1**
Na, mmol/L	141 ± 0.2	141 ± 0.4

Data are mean ± SEM. *P < 0.05; **P < 0.001; ***P < 0.0001 vs normal rats.

Administration of iron had no effect on plasma creatinine and urea at any time point (data not shown). Some mortality was observed within hours after intravenous iron (3 in the IIM group and 3 in the FCM group in the normal rats, and 1 in the IIM group of uremic rats). One uremic rat died after vehicle injection.

### Effect of intravenous iron administration on iron parameters in normal and uremic rats

**Figure 1:**
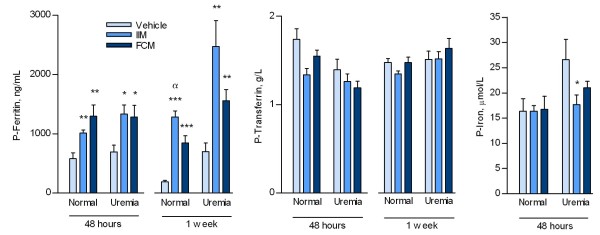
**Plasma ferritin, transferrin and iron levels in normal and uremic rats at 48 hours and 1 week after a single injection of either vehicle, iron isomaltoside (IIM) or ferric carboxymaltose (FCM).** The iron load was confirmed by significant increases of plasma ferritin levels in both normal and uremic rats receiving IIM and FCM. Mean ± SEM. *p < 0.05 vs vehicle, **p < 0.01 vs vehicle, ***p < 0.001 vs vehicle, ^α^p < 0.05 vs FCM.

Iron administration was confirmed by significant increases in plasma ferritin levels. Thus, at 48 hours both normal and uremic IIM and FCM treated groups had significantly increased plasma ferritin vs vehicle groups (p < 0.001) (Figure 1). No differences were seen in plasma ferritin when comparing IIM and FCM groups in both normal and uremic rats. No change was seen in plasma iron in either normal or uremic rats at 48 hours. In normal rats a slight decrease was seen in plasma transferrin in the IIM group vs the vehicle group (p < 0.05), whereas no change was seen in the FCM group. In the uremic rats no change was seen in plasma transferrin levels.

At 1 week the plasma ferritin increased significantly in both normal and uremic IIM and FCM treated groups as compared to vehicle group (p < 0.01), and further the plasma ferritin was higher in the normal IIM group when compared to the normal FCM group (Figure 1, p < 0.05).

### Effect of intravenous iron administration on plasma PTH

The uremic rats had significantly elevated plasma PTH levels as compared to normal rats (Table 1). No significant changes were seen in plasma PTH levels after intravenous iron or vehicle in any of the groups. In normal rats in the 48 hour study, PTH levels in the vehicle group were 110 ± 38 at baseline vs 82 ± 32 pg/mL at 48 hours, in the IIM group 47 ± 15 vs 60 ± 35 pg/mL, and in the FCM group 82 ± 39 vs 60 ± 23 pg/mL. In the 1 week study plasma PTH in the vehicle group was 18 ± 2 at baseline vs 16 ± 2 pg/mL at 1 week, in the IIM group 30 ± 7 vs 21 ± 3 pg/mL, and in the FCM group 40 ± 14 vs 26 ± 6 pg/mL.

Similarly, in uremic rats no significant changes in plasma PTH were observed: In the 48 hour study, plasma PTH in the vehicle group was 1452 ± 516 at baseline vs 1567 ± 664 pg/mL at 48 hours; in the IIM group 297 ± 81 vs 645 ± 344 pg/mL; and in the FCM group 466 ± 161 vs 911 ± 326 pg/mL. Plasma PTH in the 1 week study of uremic rats was stable; in the vehicle group 2477 ± 388 at baseline vs 2596 ± 464 pg/mL at 1 week; in the IIM group 2609 ± 282 vs 2391 ± 400 pg/mL; and in the FCM group 2243 ± 264 vs 1884 ± 344 pg/mL. Plasma Ca^2+^ was measured at all intermediate time points and remained stable within 48 hours and 1 week in both normal and uremic rats (data not shown).

### Effect of intravenous iron administration on iFGF23 and plasma phosphate in normal and uremic rats

**Figure 2:**
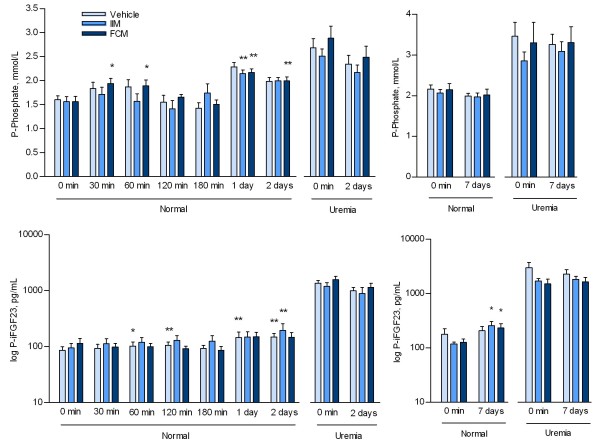
**Plasma phosphate and intact FGF23 in normal and uremic rats at 48 hours (a + b) and 1 week (c + d) after vehicle or a single high dose of intravenous iron isomaltoside (IIM) or ferric carboxymaltose (FCM).** Compared to vehicle, intravenpous iron had no significant effect on plasma phosphate and iFGF23 levels in normal and uremic rats, neither within the first 48 hours nor 1 week after the injection. Within the single group some significant variations in both phosphate and iFGF23 levels were observed, but not different from what was observed in the vehicle group. Mean ± SEM. *p < 0.05 vs 0 minutes, **p < 0.01 vs 0 minutes within the groups.

Plasma iFGF23 and phosphate were measured at several time points within 48 hours after intravenous iron administration in normal rats and at baseline and 48 hours in uremic rats. No significant differences were found between vehicle, IIM or FCM-groups at any time in neither normal nor uremic rats. Significant variations in both plasma phosphate and iFGF23 were however observed within the single group during the experiments. In the groups of normal rats plasma iFGF23 and phosphate increased, but inconsistently within 48 hours and at different time points (Figure 2). In the uremic rats both plasma phosphate and iFGF23 levels were significantly elevated as compared to the normal groups (Table 1, p < 0.001). No significant differences were found between the vehicle group and the two iron groups in plasma iFGF23 and plasma phosphate at baseline. After 48 hours a slight decrease was seen in both phosphate and iFGF23 levels with no difference between the groups (Figure 2 a + b). In a subgroup of uremic rats measurements were performed at intermediate time points as well, again with no differences between the vehicle group and the two iron groups at any time (data not shown).

As no significant differences in iFGF23 levels were observed at any time between the groups within 48 hours, six additional groups of normal and uremic rats had similar doses of iron or vehicle injected and were followed for 1 week. In the 1-week study no change was seen in plasma phosphate levels (Figure 2 c + d). iFGF23 levels in normal rats increased significantly (p < 0.05) in the two iron treated groups, as compared to their baseline; this increase was however not significantly different from that of the control group, and as such no difference in response was seen between the 3 groups. In the uremic rats no significant changes were seen in neither plasma phosphate nor iFGF23 after 1 week.

### The stability of intact FGF23 in contrast to C-terminal FGF23 during short-term experimental conditions

**Figure 3:**
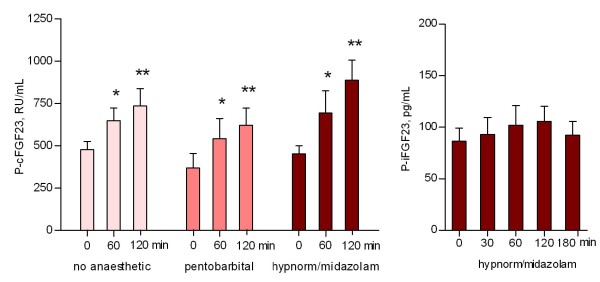
**Stability of plasma intact FGF23 levels as compared to increasing C-terminal FGF23 during short term experimental conditions.** Monitoring of iFGF23 resulted in stable levels, while levels of cFGF23 increased significantly already within 1 hour (p < 0.05), and remained elevated for 2 hours (p < 0.01). This increase of cFGF23 was seen both in non-anesthetized, awake rats and anesthetized rats and was similar for the two different anesthetics used. The rats were not subject to further intervention. Mean ± SEM. *p < 0.05 vs 0 min, **p < 0.01 vs 0 min.

The plasma levels of cFGF23 were examined by repeated measurements in three groups of normal rats, one witout anesthesia, and two groups anesthetized either with pentobarbital or hypnorm/midazolam, but still without any other intervention. Already after 1 hour did the levels of cFGF23 increase significantly (p < 0.05), and remained elevated after 2 hours (p < 0.01). This increase was seen in both non-anesthetized and anesthetized rats and was similar for the two different anesthetics used. The levels of iFGF23 were in contrast to cFGF23 levels, stable during the first 2 hours of experimental time (Figure 3). Therefore, only iFGF23 was chosen to be measured in the present study.

## Discussion

This is the first study to examine the effect of intravenous iron on plasma iFGF23 levels in non-iron depleted rats. The effect of a single high dose of two different iron preparations intravenously, iron isomaltoside 1000, and ferric carboxymaltose, as compared to that of saline was examined. The dose of the two iron preparations given in this trial was 80 mg/kg body weight. For comparison, the maximal recommended single dose in humans of IIM is 20 mg/kg body weight and a total of 1000 mg for FCM. The biological effectiveness was ensured by significantly elevated plasma ferritin levels. No effect of very high doses of iv iron was found on iFGF23 neither in normal rats nor in uremic rats in both short-term (two days) and long-term (one week) studies.

Experimental uremia was associated with severely disturbed mineral homeostasis, with significant hyperparathyroidism, hyperphosphatemia, hypocalcemia and very high levels of FGF23. Previously, it has been shown that the increased levels of FGF23 in uremia depend upon the degree of secondary hyperparathyroidism [29] and that a feed-back mechanism probably exists with FGF23 also having a regulatory effect on parathyroid function [30]. Intravenous iron administration did in the present investigation not result in a further increase of FGF23 and did not induce changes in plasma PTH, phosphate or Ca^2+^ levels. Blood samles were obtained at close intervals and as such, even a staggered response to iv iron of some of the parameters involved in the integrated regulation between FGF23 and PTH should have been detected.

Studies on genetic hypophosphatemic disorders indicate the existence of a robust link between iron deficiency and FGF23, although the exact regulatory mechanism is not known. It was in the hypophosphatemic disorders, autosomal dominant hypophosphatemic rickets (ADHR) and tumor induced osteomalacia (TIO), that FGF23 first was identified and its role in phosphate metabolism recognized [31,32]. ADHR, TIO, X-linked hypophosphatemic rickets (XLH) and autosomal recessive hypophosphatemic rickets (ARHR) are all characterized by excess of iFGF23 [33]. These disorders show similar phenotypes characterized by hypophosphatemia, low 1,25(OH)_2_D levels and osteomalacia, but unlike XLH and ARHR, ADHR shows incomplete penetrance and variable age of onset. In ADHR, FGF23 is resistant to proteolytic cleavage due to mutations in the area of the cleavage site, separating the N-terminal from the C-terminal part [31]. Studies of families with ADHR have led to the observation that the onset of disease in ADHR-subjects can occur during physiological states of iron-deficiency [34]. These observations, linking iron status to FGF23, have later been supported by studies in ADHR knock-in mouse, where iron deficiency was sufficient to cause the ADHR phenotype [22].

Previous observations suggested that administration of parenteral iron might be associated with hypophosphatemia and osteomalacia [14,26]. Schouten et al. therefore examined the effect of a single infusion of iron polymaltose in non-CKD patients suffering from iron deficiency, and found a significant and prolonged increase in iFGF23 levels, which was accompanied by a decrease in plasma phosphate, increased phosphate excretion and suppressed 1,25(OH)_2_D [13]. The mechanisms underlying these changes are still speculative, although it has been suggested that iron might be involved in the regulation of the rate of enzymatic cleavage of iFGF23, putting forward the idea, that increased iron levels inhibit and iron deficiency increases protease activity which would fit with the findings of elevated cFGF23 fragments in patients with low plasma ferritin. In the present study, however, no increase in iFGF23 was observed and no changes in plasma phosphate levels were induced by any of the two iron compounds given intravenously neither in short-term nor in long-term investigations.

The results of the present investigation are interesting in relation to some recently published results in human subjects, where a single dose of ferricarboxymaltose (FCM) corresponded with an increase in iFGF23 after two days and one week and resulted in increased fractional excretion of phosphate and decreased plasma phosphate, while iron dextran in the same study did not affect iFGF23 levels [11]. A main difference to our study, beside the obvious difference in species, is that the human subjects were severely iron depleted [11], while the rats in the present study not were iron deficient.

A further small number of human studies have examined the effect of a single iv iron dose on FGF23 and phosphate levels and reported varying results. Two studies found an increase of plasma iFGF23 within the first week after iv iron [10,13], while one study, examining saccharated ferric oxide, found unchanged iFGF23 levels during the first week and then a rise after three weeks of repeated weekly administration [12]. In another study, using a cFGF23 assay and a single dose of FCM in 47 non-dialysis CKD patients with iron deficiency it was found that both plasma phosphate and cFGF23 were reduced for up to 12 weeks [9].

It should be stressed that all these human studies were performed in iron-deficient subjects and that the physiological response to iron load could differ according to baseline iron status. Our purpose was however to examine the effect of intravenous iron in iron repleted conditions and doing so no effect on iFGF23 was found and the phosphate levels were stable. In a study relating iron levels with iFGF23 and cFGF23 in both ADHR and healthy subjects Imel et al. found that the iron-levels in ADHR subjects were negatively correlated to both iFGF23 and cFGF23. In healthy subjects they found no correlation between iron levels and iFGF23, while a negative correlation to cFGF23 made them hypothesize, that iron deficiency might cause increased expression of FGF23, which in healthy subjects is counter regulated by an increased cleavage [23].

When iv iron is administered, the iron-carbohydrate agents enter the reticuloendothelial system (RES). Then iron is subsequently released, and either bound to intracellular ferritin or transferrin in the blood. The rate of uptake into RES depends on the particle size, with small molecules being taken up rapidly. Only a small fraction of iron is directly transferred to transferrin from the iron-carbohydrate particle. The release of iron from the RES is dependent on iron-status. In anemic, iron-deficient individuals iron is rapidly released from the RES and it has been reported that all iron is incorporated into erythrocytes within 2–4 weeks in severely anemic, iron deficient individuals [35]. Furthermore, also the carbohydrate part of the iron agents could interfere with FGF23 synthesis and degradation [11], and as such, apparently contradictory results could be due to the use of different iron agents.

The mechanism behind the effect of iv iron on both iFGF23 and cFGF23 is still unresolved. In normal rats, without any intervention, the cFGF23 levels turned out to be significantly increasing within a brief experimental time period of 120 minutes. This was in contrast to levels of iFGF23, which remained stable. The behavior of cFGF23 did not depend upon the use of anesthetics. As such, we concluded that in this experimental rat model measurements of cFGF23 were not suitable for studying the rapid regulation of FGF23. In the present study therefore only the biologically active iFGF23 was measured. Thus, an eventual effect of iron on the metabolism of FGF23 was not studied, although such a possibility recently has been proposed [13]. Whether the observed increase in cFGF23 might be due to a diurnal variation [36-39] must await future studies.

An apparent rise in iFGF23 levels was seen normal rats after 48 hours and 1 week in both the two iron groups as well as in the vehicle group, but with no significant differences between the three groups, indicating that this rise was not caused by the iron administration. iFGF23 levels did not increase in any of the uremic groups. These results underline the importance of including an appropriate control group, when evaluating FGF23 levels.

## Conclusion

In conclusion, a single intravenous high dose of either iron isomaltoside 1000 or ferric carboxymaltose had no significant effect on intact FGF23 plasma levels for up to 7 days in non-iron deficient normal and uremic rats.

## Competing interests

The authors declare that they have no competing interests.

## Authors’ contributions

EG: Conception, design, analysis and interpretation of data, drafting the article. JH-B: Analysis and interpretation of data. MLM: Analysis and interpretation of data. EL: Conception and design, and revising article. KO: Providing intellectual input of critical importance to the work and revising the article. All authors read and approved the final manuscript.

## Pre-publication history

The pre-publication history for this paper can be accessed here:

http://www.biomedcentral.com/1471-2369/14/281/prepub
